# Physical exercise and social media addiction among adolescents: a chain mediation analysis of cognitive flexibility and negative attention bias

**DOI:** 10.3389/fpsyt.2026.1944377

**Published:** 2026-09-03

**Authors:** Jie Luo, Shiqi Long, Yuhan Chen, Mengting Pan, Yang Liu, Xiaodi Ma, Jianzhang Luo

**Affiliations:** 1 College of Physical Education of CDU, Chengdu University, Chengdu, China; 2 School of Physical Education and Arts, Hunan University of Medicine, Huaihua, China; 3 Jishou University, Jishou, China; 4 Southwest University, Chongqing, China; 5 Heilongjiang University of Chinese Medicine, Harbin, China

**Keywords:** adolescents, cognitive flexibility, negative attention bias, physical exercise, social media addiction

## Abstract

**Background and objectives:**

Physical Exercise (PE) is associated with multiple aspects of adolescent psychological and cognitive functioning. Cognitive Flexibility (CF) and Negative attention bias (NAB) may help explain the association between PE and Social Media Addiction (SMA), yet their joint indirect roles remain insufficiently examined. This study tested a cross-sectional serial indirect-effects model linking PE, CF, NAB, and SMA among Chinese adolescents.

**Methods:**

A self-reported survey was conducted among 3,375 adolescents in China, collecting data on PE, SMA, CF, NAB, and demographic variables. As an initial step, descriptive statistics and correlation analyses were conducted to examine PE, SMA, CF, and NAB. A chain mediation model was subsequently established to investigate whether CF and NAB sequentially mediated the association between PE and SMA.

**Results:**

The correlation analysis revealed that PE was significantly and negatively associated with both SMA and NAB, while exhibiting a significant positive association with CF. After incorporating CF into the model, the negative correlation between PE and SMA weakened but remained significant. In addition, CF was significantly and negatively associated with SMA, providing preliminary support for its potential mediating role in the association between PE and SMA. CF was also significantly and negatively related to NAB, whereas NAB exhibited a significant positive association with SMA. These results indicate that CF may function as an important intermediary mechanism linking PE to lower levels of SMA, potentially through its influence on NAB.

**Conclusion:**

PE was associated with lower levels of SMA both directly and indirectly, with the indirect effect operating through increased CF and reduced NAB. This serial mediation mechanism provides a more comprehensive understanding of how PE may influence adolescent mental health. The findings further highlight the potential value of PE as a preventive and intervention-oriented strategy for reducing SMA among adolescents. It is recommended that promoting PE could enhance CF and improve attention biases, thereby effectively mitigating the risk of SMA.

## Background

With the rapid expansion of digital technologies, social media has become a central component of adolescents’ social interactions and leisure pursuits, profoundly shaping their daily lives. While social media offers numerous conveniences, excessive use may have detrimental effects on cognitive and behavioral abilities, among other aspects of adolescent development (1, 2). By 2023, global social media users had reached nearly 5 billion, with approximately 1.03 billion users in China alone (3). Studies conducted among Chinese adolescents have reported that the prevalence of social media addiction (SMA) varies between 5% and 20% (4). Although social media platforms facilitate access to information, broaden adolescents’ cognitive horizons, and satisfy their needs for social interaction and entertainment, their extensive use may also entail potential risks (5). Nevertheless, the highly engaging and entertainment-oriented features of social media platforms may encourage excessive use, while exposure to content of uneven quality may contribute to distorted value formation and adversely affect adolescents’ physical health, psychological well-being, and cognitive development (6). The World Health Organization has further indicated that lifestyle and health-related behaviors account for approximately 60% of the determinants of human health, and that harmful behavioral patterns may interfere with normal growth and development (7). As highly open platforms, social media are saturated with information that can be detrimental to health. Prolonged immersion in these platforms may not only impact academic performance and individual adaptability but also pose risks to physical health, mental well-being, and cognitive development (8–10). Adolescents represent the future of any nation, and their physical and mental development warrants significant attention. Accordingly, problematic social media use among adolescents should be regarded not merely as an individual developmental concern but also as a broader public health and social issue with potential long-term consequences. Greater attention and evidence-based intervention strategies are therefore warranted.

As a relatively new manifestation of problematic internet use, SMA has attracted increasing scholarly attention within psychological research (11). SMA is characterized by a maladaptive reliance on social media platforms that may progressively manifest as symptoms commonly associated with behavioral addiction (12). Accumulating evidence indicates a significant relationship between physical exercise (PE) and SMA among adolescents (13–15). PE not only facilitates communication and interaction among students, enhances individual cognitive abilities, and reduces attention to negative stimuli, but also decreases social media usage. A more comprehensive understanding of the potential pathways linking PE to SMA among adolescents may provide valuable theoretical insights and inform the development of evidence-based prevention and intervention strategies.

Adolescence represents a crucial stage of physiological maturation and psychological development, during which PE contributes substantially to healthy growth and overall well-being. PE (16) refers to various bodily activities that expend energy through skeletal muscle activity. Existing evidence indicates that regular PE not only significantly improves adolescents’ cardiorespiratory fitness and musculoskeletal health but also enhances their mental well-being (17, 18). These psychological benefits may, in turn, reduce the risk of excessive social media use driven by emotional regulation difficulties (19). At the neurobiological level, regular participation in physical exercise (PE) may induce structural and functional changes in the prefrontal cortex, which plays a key role in executive control, decision-making, and response inhibition (20). This neuroplasticity may strengthen adolescents’ ability to regulate their screen time autonomously, thereby reducing the likelihood of SMA (21). Accordingly, Hypothesis H1 proposed that PE would be negatively associated with SMA.

In a rapidly changing developmental environment, some adolescents are able to adapt flexibly to complex learning tasks and social conflicts, quickly adjusting their strategies, while others may become trapped in rigid thinking patterns (22). These interindividual variations may be partly attributable to Cognitive Flexibility (CF), an important concept in developmental psychology. CF denotes the ability to modify attentional focus and behavioral responses in accordance with shifting task demands, rules, or priorities, as demonstrated in task-switching situations (23). The executive function framework (24) proposes that participation in PE may facilitate the development of executive functioning among adolescents. Participation in PE frequently requires individuals to make rapid decisions, modify behavioral strategies, and coordinate multiple tasks concurrently. These cognitive demands may strengthen the brain’s capacity for information processing and adaptation to complex situations, thereby facilitating the development of CF. Greater CF may, in turn, contribute to more effective regulation of SMA-related use patterns among adolescents (9). Previous evidence indicates that individuals with higher levels of CF display lower P3 amplitudes when exposed to social media notifications, suggesting stronger inhibitory control and a greater capacity to filter task-irrelevant information (25). Hypothesis H2 therefore proposed that PE would show an indirect statistical association with SMA through CF.

In environments characterized by information overload, adolescents’ brains often prioritize the processing of negative stimuli, a cognitive tendency referred to as negative attention bias (NAB) (26, 27). This bias reflects a differential allocation of attentional resources, whereby individuals exhibit heightened sensitivity to negative stimuli related to their cognitive schemas or personal experiences, leading to preferential processing. Recent empirical evidence has identified a significant relationship between PE and NAB (28). According to the attentional control theory (29), adolescents engaging in PE can enhance goal-directed attentional control, reducing interference from negative information and facilitating the reallocation of attention toward current tasks or objectives. During adolescence, individuals are generally more sensitive to external stimuli, and the complex and often negative content on social media is particularly likely to capture their attention (30). Further research indicates that excessive focus on negative information may drive adolescents to more frequently seek updates on social media or escape into virtual environments as a coping strategy, potentially fostering dependency or addictive behaviors (31). Hypothesis H3 therefore proposed an indirect statistical association between PE and SMA through NAB.

CF is an important determinant of information-processing efficiency during adolescence, particularly in the regulation of NAB (32). Empirical findings further indicate that individuals with greater CF may be better able to regulate attentional allocation when confronted with negative stimuli (33). Neuroplasticity studies indicate that systematic cognitive training tailored to changing task demands may enhance functional coupling between the prefrontal cortex and limbic structures. This not only reduces adolescents’ susceptibility to negative emotional stimuli but also enhances their psychological resilience and CF (34). PE plays a pivotal role in this process. Activities such as badminton, which are competitive in nature, not only improve adolescents’ focus and attention control but also stimulate the release of neurotransmitters like dopamine. These substances contribute to enhanced mood and psychological well-being. Through its physical and psychological benefits, engagement in PE may facilitate adolescents’ attentional disengagement from negatively valenced information, which could attenuate their responses to adverse social media content and be associated with less frequent and shorter periods of social media use (35, 36). Hypothesis H4 therefore proposed a sequential indirect association represented by PE → CF → NAB → SMA.

**Figure 1:**
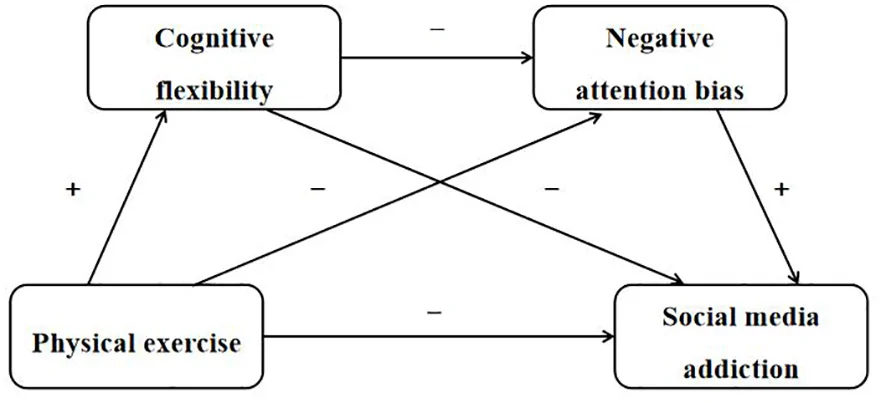
Chain mediation hypothesis model. “+” indicates a possible positive association, whereas “−” indicates a possible negative association.

Although previous studies have examined the independent associations between PE and SMA, the psychological mechanisms underlying this relationship remain insufficiently understood. The present study extends existing research by integrating CF and NAB into a unified serial mediation framework. Specifically, this model proposes that PE may be associated with improved CF, which subsequently contributes to more adaptive attentional processing by reducing NAB, ultimately providing a more comprehensive explanation of how PE relates to SMA among adolescents. By examining these sequential psychological pathways, the current study moves beyond examining isolated associations and contributes to a deeper understanding of the cognitive mechanisms involved in adolescent social media-related behaviors. **Figure 1** illustrates the proposed research model.

## 2 Methods

### 2.1 Participants

In June 2026, participants were recruited through convenience sampling from five schools located in Hunan, Sichuan, and Guangdong Provinces, China. Before administering the survey, trained researchers explained the completion procedures and relevant requirements to all participants. Questionnaires were distributed and collected in person using a paper-and-pencil format. Of the 3,375 questionnaires initially administered, 3,236 were retained after data screening, yielding a valid response rate of 95.88%. This mainly involved cases with any missing values, those who failed the lie-detection questions hidden in the items, and those with regular responses. The final sample comprised 1,600 boys (49.44%) and 1,636 girls (50.56%). Participants were between 10 and 19 years of age, with a mean age of 14.82 years (SD = 1.409).

### 2.2 Materials and measures

#### 2.2.1 Physical exercise

PE was assessed using the revised PE Level Scale developed by Liang Deqing et al. (37). This instrument evaluates three components of PE: intensity, duration, and frequency. The revised scale contains three items, and the overall score is calculated using the following equation: intensity × (duration − 1) × frequency. Higher total scores represent greater levels of PE. In the present sample, the scale demonstrated acceptable internal consistency, with a Cronbach’s alpha coefficient of 0.706.

#### 2.2.2 Social media addiction

SMA was evaluated using the Bergen SMA Scale (4). The instrument contains six items corresponding to six addiction-related domains: salience, mood modification, tolerance, withdrawal, conflict, and relapse (38). Each item is scored on a five-point Likert scale ranging from 1 (rarely) to 5 (very often). The item scores are summed to generate an overall score, with higher values reflecting more severe SMA. In the current sample, the scale exhibited good internal consistency, as indicated by a Cronbach’s alpha coefficient of 0.810.

#### 2.2.3 Cognitive flexibility

CF was measured using the CF Subscale of the Adolescent Executive Function Scale developed by Huang Chunhui et al. (39). The subscale comprises eight items, each evaluated on a three-point Likert scale ranging from 1 (often) to 3 (never). Item scores are aggregated to produce a total score, with higher values representing greater CF. In the present sample, the subscale showed good internal consistency, with a Cronbach’s alpha coefficient of 0.855.

#### 2.2.4 Negative attention bias

NAB was assessed using the corresponding subscale of the Positive and Negative Information Attention Scale. The original instrument was developed by Noguchi et al. (40) and subsequently revised by Dai Qin and Feng Zhengzhi (41). This subscale contains 10 items, each rated on a five-point Likert scale from 1 (strongly disagree) to 5 (strongly agree). The responses are summed to obtain an overall score, with higher scores reflecting a greater tendency toward NAB. Analysis revealed that the Cronbach’s α coefficient of the scale was 0.888.

### 2.3 Statistical analysis

The analytical procedures comprised tests for common method bias, evaluations of reliability and validity, descriptive statistical analysis, Pearson correlation analysis, and regression analysis. All statistical analyses were performed using SPSS version 26.0 and Hayes’ PROCESS macro. A serial mediation analysis was conducted using PROCESS Model 6, with CF and NAB specified as sequential mediators between PE and SMA. The indirect effects were estimated using bias-corrected bootstrap procedures with 5,000 resamples. Statistical significance of the indirect effects was determined based on whether the bias-corrected 95% bootstrap confidence intervals excluded zero.

## 3 Results

### 3.1 Common method deviation test

The possibility of common method bias was evaluated using Harman’s single-factor procedure. An unrotated exploratory factor analysis identified four factors with eigenvalues exceeding 1. The first factor accounted for 20.01% of the total variance, which was substantially lower than the recommended threshold of 40%. These findings indicate that common method bias was unlikely to pose a substantial threat to the present results.

### 3.2 Description analysis

The results presented in **Table 1** indicate significant differences in PE, SMA, CF, and NAB across gender, only-child status, and academic grade. Additionally, PE and SMA showed significant differences based on living conditions, while PE exhibited a significant difference in relation to school residence status (see **Table 1**).

**Table 1:** Descriptive statistics and group comparisons by demographic characteristics.

Variables		PE		SMA		CF		NAB	
		M	SD	M	SD	M	SD	M	SD
Gender	Boys	19.96	21.71	13.79	5.15	18.26	3.91	29.48	9.06
	Girls	14.06	15.88	14.64	4.86	17.79	3.85	30.78	8.90
	t	8.809***		-4.843***		3.450**		-4.089***	
Only child status	Only children	23.87	22.71	13.42	5.17	18.60	3.83	28.77	9.08
	Not only children	16.06	18.50	14.33	4.99	17.94	3.89	30.32	8.98
	t	6.454***		-3.357**		3.097**		-3.163**	
Live on campus or not	Live on campus	16.17	17.84	14.25	5.00	17.99	3.96	30.13	9.04
	Not live on campus	18.52	21.50	14.16	5.08	18.07	3.74	30.14	8.93
	t	-3.120**		0.492		-0.551		-0.017	
Place of Residence	Towns	18.83	20.95	13.98	5.00	18.12	3.86	30.02	9.194
	Village	15.22	17.22	14.45	5.04	17.93	3.90	30.25	8.813
	t	5.344***		-2.669**		1.421		-0.715	
Grade	Grade 5	15.05	23.83	13.97	3.66	17.65	3.32	29.95	8.91
	Grade 6	14.76	19.89	14.11	4.78	18.01	4.00	30.51	8.64
	Grade 7	16.93	20.44	14.05	4.97	18.37	3.89	29.76	9.47
	Grade 8	22.73	19.29	13.58	5.03	18.78	3.96	29.05	9.56
	Grade9	32.00	22.34	14.41	5.08	18.17	3.73	29.98	9.05
	Grade 10	12.49	15.57	14.53	5.15	17.65	4.03	30.30	8.89
	Grade 11	16.09	20.05	14.55	4.83	17.51	3.31	31.54	7.92
	Grade 12	23.61	30.69	13.35	4.75	19.74	3.73	27.70	10.44
	F	32.753***		3.033**		8.402***		4.073***	

**p<0.01; ***p<0.001.

### 3.3 Correlation analysis

As presented in **Table 2**, PE was inversely associated with SMA and NAB, whereas it showed a positive association with CF. SMA was negatively related to CF but positively related to NAB. In addition, a significant inverse correlation was observed between CF and NAB.

**Table 2:** Correlation analysis.

Variables	1	2	3	4
1 PE	–			
2 SMA	-0.118***	–		
3 CF	0.130***	-0.237**	–	
4 NAB	-0.096***	0.167**	-0.374**	–

**p<0.01; ***p<0.001.

### 3.4 Mediation models

As shown in **Table 3**, PE was negatively associated with SMA and NAB, but positively associated with CF. After CF and NAB were simultaneously included in the model, the association between PE and SMA decreased in magnitude from β = −0.093 to β = −0.066, although it remained statistically significant. CF was also negatively associated with SMA, a pattern consistent with a partial mediating role of CF. Moreover, CF was negatively associated with NAB, whereas NAB was positively associated with SMA. Taken together, these associations provide preliminary support for a serial indirect pathway linking PE to SMA through CF and NAB.

**Table 3:** Tests the mediation model.

Outcome variable	Predictive variable	β	SE	t	R²	F
SMA	PE	-0.093	0.018	-5.168***	0.025	11.694***
CF	PE	0.106	0.018	5.935***	0.030	14.111***
NAB	PE	-0.037	0.017	-2.199*	0.146	68.744***
	CF	-0.364	0.017	-22.038***		
SMA	PE	-0.066	0.018	-3.786***	0.077	29.715***
	CF	-0.188	0.018	-10.183***		
	NAB	0.083	0.018	4.517***		

*p<0.05; ***p<0.001.

**Figure 2:**
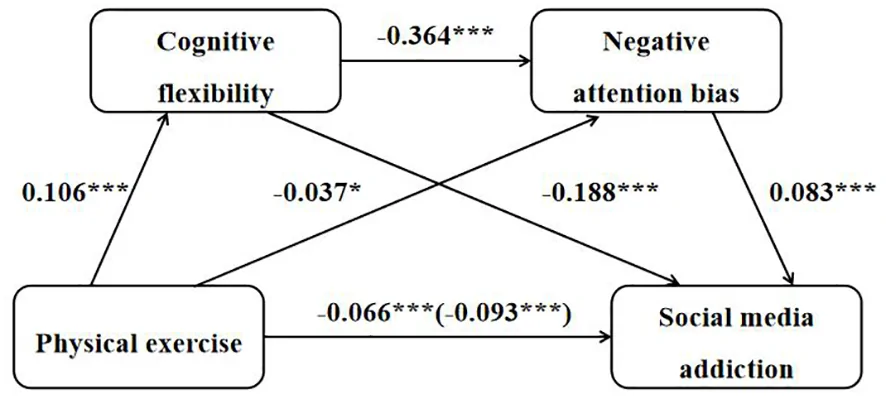
Diagram of the chain mediation model (standardized regression coefficients are presented. Covariates were statistically controlled).

As reported in **Table 4**, the total effect of PE on SMA was −0.093. The direct effect was −0.066, accounting for 70.97% of the total effect. The remaining 29.03% was attributable to the indirect effect through CF and NAB. Because the confidence intervals for both the direct and indirect effects did not include zero, the results support a statistically significant partial mediation effect. The results are shown in **Figure 2**.

**Table 4:** Path analysis of mediation model.

Intermediate path	Effect size	SE	Bootstrap 95% CI	Proportion of mediating effect
Total association	-0.093	0.018	-0.128, -0.058	100%
Conditional direct association	-0.066	0.018	-0.101, -0.032	70.97%
Total indirect effect	-0.026	0.005	-0.036, -0.017	29.03%
PE → CF → SMA	-0.020	0.004	-0.028, -0.012	21.52%
PE → NAB → SMA	-0.003	0.002	-0.007, 0.001	4.28%
PE → CF → NAB → SMA	-0.003	0.001	-0.005, -0.002	3.23%

## 4 Discussion

The present findings demonstrated a significant association between PE and SMA among adolescents, supporting Hypothesis H1. Rather than directly replacing problematic digital behaviors, PE may provide adolescents with alternative opportunities for offline engagement, social interaction, and emotional regulation, thereby weakening excessive reliance on social media platforms (14, 15, 42). Regular participation in PE may facilitate face-to-face communication and strengthen real-world social connections, which could reduce adolescents’ motivation to seek social reinforcement primarily through online environments (43). Therefore, schools should consider promoting diverse and enjoyable PE programs to create supportive activity environments and encourage sustainable exercise habits among adolescents.

The mediation analysis supported Hypothesis H2, indicating that CF may represent an important psychological pathway underlying the association between PE and SMA. Adolescents who engage more frequently in PE may develop enhanced CF through repeated demands for decision-making, strategy adjustment, and adaptive responses during physical activities. Previous neuroimaging evidence suggests that PE is associated with increased activation in brain regions involved in executive functioning, including the prefrontal and parietal cortices, which may contribute to improvements in cognitive regulation abilities (44). Enhanced CF may enable adolescents to better evaluate online stimuli, regulate impulses, and flexibly adjust their social media-related behaviors when encountering digital temptations (45). These findings suggest that interventions aimed at adolescent SMA may benefit from integrating PE with cognitive training approaches targeting executive control rather than focusing solely on increasing exercise participation.

Consistent with Hypothesis H3, the present study further revealed that NAB may serve as another pathway linking PE with SMA. Adolescents with insufficient PE participation may exhibit greater vulnerability to negative information processing tendencies, which could influence emotional experiences and coping behaviors. According to the cognitive-behavioral framework (46), excessive attention toward negative information may increase emotional distress and encourage individuals to seek relief or compensation through online platforms. PE may contribute to attentional regulation by improving individuals’ ability to disengage from negative stimuli and redirect attention toward goal-relevant information. Therefore, PE-based interventions may have potential value in improving attentional processing patterns and promoting healthier digital behaviors among adolescents.

Furthermore, the serial mediation analysis supported Hypothesis H4, revealing a sequential pathway involving PE, CF, NAB, and SMA. Specifically, enhanced CF may facilitate more adaptive allocation of attentional resources, thereby reducing the tendency to focus excessively on negative information. This interpretation is consistent with previous research suggesting that cognitive flexibility plays an important role in regulating attentional processes and emotional responses (47). The findings extend previous research by suggesting that the relationship between PE and adolescent SMA may involve a combination of executive functioning and attentional mechanisms rather than a single psychological pathway. Accordingly, future intervention programs may benefit from combining PE with psychological strategies that strengthen cognitive flexibility, attentional control, and information literacy.

In summary, enhancing PE contributes to the improvement of CF in adolescents, which in turn reduces the bias in their attention toward negative information, thereby lowering the risk of SMA. Therefore, the prevention and treatment of adolescent SMA require not only external environmental support and guidance but also effective monitoring and management of the individual’s internal psychological changes.

### 4.1 Limitations

This study has several limitations. First, the use of data collected at a single time point precludes conclusions about the causal direction or temporal ordering of the associations among PE, CF, NAB, and SMA. Subsequent research could employ longitudinal, cross-lagged panel, or experimental approaches to clarify the temporal direction of these relationships and determine whether they remain stable over time. Second, the proposed model did not include potentially important moderating or contextual factors, such as school connectedness, bullying experiences, parenting practices, and peer support. Incorporating these boundary conditions may improve the explanatory scope of the theoretical framework. Third, the sample characteristics restricted the examination of demographic and cultural heterogeneity. Subsequent studies could enhance sample size and heterogeneity and examine whether the observed associations differ by gender, age, socioeconomic status, geographic region, and cultural background. Finally, the exclusive use of self-reported data may have rendered the findings susceptible to recall errors, socially desirable responding, and bias arising from the use of a single measurement method. Subsequent research could combine self-report instruments with objective measures, including accelerometer-derived indices of PE, digitally recorded patterns of social media use, and behavioral tasks assessing CF and NAB.

## 5 Conclusion

In conclusion, PE was significantly and inversely associated with SMA among adolescents. The mediation analysis identified three statistically significant indirect associations involving CF and NAB: PE → CF → SMA, PE → NAB → SMA, and the sequential pathway PE → CF → NAB → SMA. Greater engagement in PE was related to enhanced CF and reduced NAB; in turn, both greater CF and weaker NAB were linked to lower SMA scores. These findings provide further evidence that CF and attentional processing may represent important psychological pathways underlying the association between PE and SMA. The proposed framework extends current understanding of the pathways linking PE with SMA among adolescents and offers empirical guidance for prevention initiatives targeting PE participation, CF, and attentional regulation.

## Data Availability

The raw data supporting the conclusions of this article will be made available by the authors, without undue reservation.
